# Integrated Care Planning for Cancer Patients: A Scoping Review

**DOI:** 10.5334/ijic.2543

**Published:** 2017-11-13

**Authors:** Anum Irfan Khan, Erin Arthurs, Sharon Gradin, Marnie MacKinnon, Jonathan Sussman, Vishal Kukreti

**Affiliations:** 1 Institute for Health Policy Management and Evaluation, University of Toronto, Toronto, ON, CA; 2 Cancer Care Ontario, Toronto, ON, CA; 3 Department of Oncology, McMaster University, Hamilton, ON, CA; 4 Princess Margaret Cancer Centre, University Health Network, Toronto, ON, CA

**Keywords:** integrated care, cancer, care planning

## Abstract

**Introduction:**

There has been a growing emphasis on the use of integrated care plans to deliver cancer care. However little is known about how integrated care plans for cancer patients are developed including featured core activities, facilitators for uptake and indicators for assessing impact.

**Methods:**

Given limited consensus around what constitutes an integrated care plan for cancer patients, a scoping review was conducted to explore the components of integrated care plans and contextual factors that influence design and uptake.

**Results:**

Five types of integrated care plans based on the stage of cancer care: surgical, systemic, survivorship, palliative and comprehensive (involving a transition between stages) are described in current literature. Breast, esophageal and colorectal cancers were common disease sites. Multi-disciplinary teams, patient needs assessment and transitional planning emerged as key features. Provider buy-in and training alongside informational technology support served as important facilitators for plan uptake. Provider-level measurement was considerably less robust compared to patient and system-level indicators.

**Conclusions:**

Similarities in design features, components and facilitators across the various types of integrated care plans indicates opportunities to leverage shared features and enable a management lens that spans the trajectory of a patient’s journey rather than a phase-specific silo approach to care.

## Introduction

Cancer patients utilize a wide range of services from multiple providers across settings at various points during their cancer journey, including oncologists, non-cancer specialists, primary care physicians, nurses, pharmacists, physiotherapists and social workers [1]. Given the high cost and complexity of their evolving needs from diagnosis to either survivorship or palliative care, cancer patients require care that is integrated across providers (medical, nursing and allied-health practitioners) and settings over time [1,2,3].

Given the diverse range of providers and settings involved in caring for cancer patients [4,5], there has been a strong emphasis on the use of care plans [6,7] to support care management in cancer patients [8,9,10,11]. Care plans are seen as tools to organize care processes [12], monitor variance and outcomes [13], facilitate communication between providers, and promote adherence to best clinical evidence [12,13,14]. However there is currently limited consensus around a definition for the concept of ‘care plans’ and related constructs including care pathways, clinical pathways, care maps, individualized care plans, and care protocols etc., which are often used interchangeably in the literature [7,15,16,17]. For the purposes of this review, the term ‘care plan’ will be used to refer to these above-mentioned terms.

Existing literature indicates that the use of care plans across a variety of diseases and settings, has helped reduce in-hospital complications [15,18], enhanced communication between providers, and improved the quality and efficiency of care [12]. But much of the existing work around care planning pertaining to cancer patients has either focused exclusively on the clinical elements of care, i.e., adherence to evidence-based guidelines around surgical and diagnostic procedures etc. [19], or has targeted individual stages in the cancer trajectory (i.e., survivorship or palliative care) [20] rather than using a patient-centered or service delivery lens to inform the integration of care across the continuum. As such little is known about how integrated care plans for cancer patients are developed and implemented, what activities they feature and which organizational and system-level factors enable their uptake.

In the absence of consensus around what constitutes an integrated care plan for cancer patients, a scoping review was conducted to explore the key components of integrated care plans, identify facilitators and barriers associated with their use and examine the indicators that have been utilized to assess their impact. Scoping reviews are commonly used to understand the existing breadth of research on a topic, identify gaps in existing literature and assess the need for further research inquiry [21].

## Methods

A cursory review of grey and peer-reviewed literature was initially conducted to better understand existing terminology and develop a definition of an ‘integrated care plan’ for cancer patients. The definition development stage involved examining systematic reviews and meta-analyses focused on clinical pathways, care plans, critical pathways (and other similar concepts) to assess how these constructs are currently defined in existing literature. Following the completion of this first stage, an initial definition was drafted and feedback was obtained through widespread consultation via a panel of key experts in cancer care, which included healthcare providers, administrators/management and researchers from across the core disciplines represented in the continuum of cancer care (i.e., primary care, nursing, and oncology). Following the working group consultation an integrated care plan for cancer patients was defined as a:

1. Structured plan of care that engages two or more providers (multi-disciplinary) or is being used in two or more settings that,
2. Involves care planning longitudinally within or across the stages of cancer (i.e., survivorship, palliative etc.), and
3. Outlines specific steps/elements of care/mechanisms, with the goal of improving patient, family and/or provider experience, and enabling greater efficiency in care delivery.

Following the development and validation of this definition for an integrated care plan by the expert panel, a scoping review was conducted to explore the core components, drivers for uptake, and key outcome measurement approaches involved in the evaluation of integrated care plans for cancer patients. The use of the term ‘integrated care plans’ for this review stems from the focus of this scoping review on cancer patients specifically. The term ‘care plans’ and ‘care planning’ are commonly used in both grey and peer-reviewed literature pertaining to the management of cancer patients [22,23,24,25]. The overall concept of care planning, and terms such as ‘advanced care planning’ and ‘care planning for end of life’, have a strong grounding in cancer literature [26,27,28]. As such the term ‘integrated care plan’ was seen as an appropriate choice to describe the organization and delivery of care for this particular patient population.

Key search terms included care map, clinical pathway, care pathway, care plans, patient care planning, critical path/pathway, individualized care plans, patient care plans, advance care planning, or patient care conferences, and cancer or neoplasms, in Medline, CINAHL, Embase, PubMed and HealthStar between March 1995 and March 2015.

All study designs were included since scoping reviews do not examine study quality [21]. The following inclusion and exclusion criterion were applied to retrieved articles:

Articles were included if the:

- Description of the integrated care plan, care/clinical pathway/protocol (or similar terms) maps onto the integrated care plan definition that has been validated by the expert panel.
- Integrated care plan was being used to manage care in adults with cancer (all disease sites were included).
- Integrated care plan was initiated following a cancer diagnosis (screening and prevention were considered to be out of scope for this review).
- Article reported on either the development process and outcomes and/or barriers and facilitators associated with integrated care plan development and/or uptake.

Articles were excluded if:

- There was no clear definition for the integrated care plan, care/clinical pathway/protocol (or similar terms) or the definition did not align with the proposed definition developed by the expert panel.
- The integrated care plan was not focused exclusively on organizing and delivering care for cancer patients or involved caring for children/youth with cancer.
- Neither outcomes nor barriers or facilitators were reported on.

Level 1 (title and abstract search) and Level 2 (full-text review) screening was conducted by two independent reviewers (AIK and EA). The data extraction tool was developed and refined by the research team following a testing phase conducted by AIK, EA and VK. Common themes around the key components, facilitators and barriers, and outcome measurement approaches were examined via a thematic analysis [29] using NVIVO (Version 11), which involved exploring emerging and recurrent themes observed across included studies [29]. Open coding was used to develop initial codes based on common ideas emerging from the extracted data. Following a refinement of the coding schema, key themes were organized into a conceptual framework. The steps guiding the conduct of this review are based on existing methodological recommendations around conducting scoping reviews [21,30] and are summarized in Table 1.

**Table 1:** Key stages and procedures used in conducting this scoping review.

Stage	Description

**1. Clarifying purpose and identifying research questions**	• Key research questions were shared with the expert panel and questions were refines to balance breadth with feasibility
**2. Identifying relevant studies**	• Development and refinement of search strategies and selection of databases
	• Testing and refinements of inclusion and exclusion criterion for screening
**3. Study selection**	• Independent application of screening criterion at two levels – title and abstract review and full article review by two reviewers (AIK and EA)
	• Resolution of disagreements by a third reviewer (VK) to determine final inclusion/exclusion
**4. Data extraction**	• Development, testing and application of the data extraction tool
**5. Data analysis**	• Summarizing descriptive characteristics of included articles
	• Thematic analysis of extracted data and assessing the implications of findings for future research and policy changes
**6. Consultation with key stakeholders**	• Development of a knowledge translation strategy to share the overall conceptual framework and findings with a broad group of stakeholders and experts for further validation

## Results

**Figure 1:**
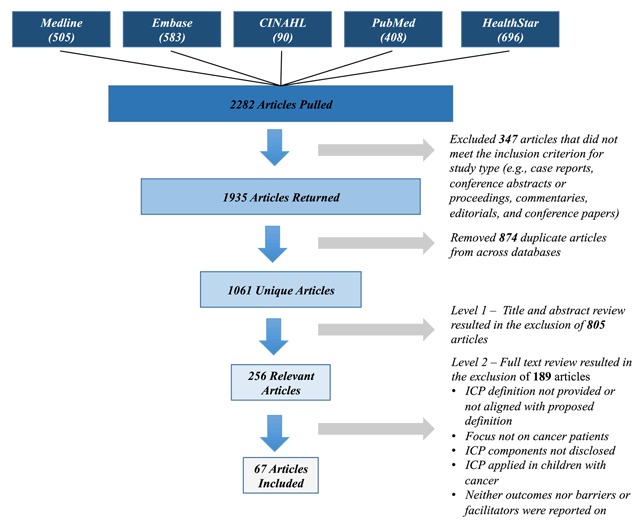
Overview of article retrieval, screening and data extraction stages.

A total of 1061 articles underwent title and abstract review (Level 1 screening), which resulted in 805 articles being excluded. Following Level 1 screening, 256 articles were included for full text review (Level 2 screening), after which 67 articles were retained for data extraction and analysis. Figure 1 provides an overview of the screening and abstraction process.

### Descriptive findings

Broadly five types of integrated care plans have been described, based primarily on the trajectory of cancer care: surgical, systemic, survivorship, palliative and comprehensive. Integrated care plans focused on organizing and delivering care during the surgical stage of cancer care were the most common type of integrated care plan (41.8%), followed by integrated care plans focused on survivorship (35.8%) and palliative (13.4%) stages of the cancer journey. Comprehensive integrated care plans, which included a transition from one stage to another, for example from treatment/surgical intervention into survivorship, represented 7.5% of the total. Only 1 article was focused on systemic treatment (1.5%). Most articles discussed the implementation of a newly developed integrated care plan, but some articles described outcomes associated with the use of an existing integrated care plan including the Journey Forward Care Plan [31] and the LIVESTRONG Care Plan [32] (both of which are focused on survivorship care) as well as the Liverpool Care Pathway [33] which is focused on palliative care.

Integrated care plans were used to manage care across multiple disease sites including breast, esophageal and colorectal cancer, with the largest proportion of integrated care plans focused on breast cancer (26.9%). Some disease sites were grouped for ease of interpretation, specifically the gynecological cancer category includes cervical, ovarian, vaginal and endometrial cancers. Articles originated from a diverse range of countries including both public and private pay systems. About 40% of articles were based in the United States, followed by the United Kingdom (10.4%) and Canada (10.4%). European countries were also well represented in the total sample with integrated care plans from Denmark, Germany, Italy, Netherlands and Sweden.

The medium in which the integrated care plan is delivered, i.e., electronic, paper or combination format, was not indicated for about half of included studies. Approximately 31% of included studies reported the delivery of the plan through a paper medium that providers and sometimes patients had access to, whereas only 9% reported the use of an electronic platform. The combination format ranged from paper copies being posted in the patients’ rooms and an electronic version being added to the patient’s electronic hospital record [34] to patients receiving a partially completed paper copy of their survivorship care plan which is iteratively adjusted over time [35]. Most studies represented a prospective observational design with no control group (37.3%), or a pre and post comparison of outcomes with a control group (31.3%). There were only 8 randomized control trials (11.9%). While study design was not accounted for in assessing articles for inclusion, the two levels of screening involved two authors (AIK and EA) independently verifying if each article met the inclusion criterion that had been validated by the expert panel. The descriptive characteristics of included articles are available in Table 2.

**Table 2:** Descriptive features of included articles.

Descriptive characteristics	Total (n = 67)	Relevant articles

**Type**		
Surgical	28 (41.8%)	[34,36,37,38,39,40,41,42,43,44,45,46,47,48,49,50,51,52,53,54,55,56,57,58,59,60,61,62]
Survivorship	24 (35.8%)	[32,35,63,64,65,66,67,68,69,70,71,72,73,74,75,76,77,78,79,80,81,82,83,84]
Palliative	9 (13.4%)	[85,86,87,88,89,90,91,92,93]
Comprehensive	5 (7.5%)	[94,95,96,97,98]
Systemic	1 (1.5%)	[99]
**Disease sites**		
Breast	18 (26.9%)	[35,45,63,65,66,67,68,69,72,73,77,79,82,83,84,95,96,98]
All	11 (16.4%)	[75,81,85,86,87,88,89,90,91,92,93]
Esophagus	7 (10.4%)	[37,47,48,49,50,52,97]
Colorectal	5 (7.5%)	[41,43,46,64,80]
Multiple^1^	3 (4.4%)	[32,76,78]
Prostate	4 (6.0%)	[44,58,62,94]
Head and Neck	4 (6.0%)	[34,36,54,61]
Gynecological^2^	5 (7.5%)	[57,60,70,71,74]
Gastric, Bladder, Lung, Pancreatic, Brain, Larynx, and Testicular	10 (14.9%)	[38,39,40,42,51,53,55,56,59,99]
**Country**		
USA	27 (40.3%)	[35,50,51,52,53,54,55,56,57,58,59,60,61,62,73,74,75,76,77,78,79,80,81,82,83,84,93]
UK	7 (10.4%)	[46,47,48,49,72,92,98]
Canada	7 (10.4%)	[34,36,37,38,65,66,67]
Netherlands	4 (6.0%)	[70,71,88,89]
Germany	4 (6.0%)	[39,40,68,69]
Denmark, Italy, Australia, Singapore, Belgium, China, Japan, Spain, Sweden, Taiwan, Turkey and Multiple^3^	18 (26.9%)	[32,41,42,43,44,45,63,64,85,86,87,90,91,94,95,96,97,99]
**Medium**		
Paper	21 (31.3%)	[38,39,47,49,55,57,59,60,61,62,65,71,74,75,78,80,82,83,85,98,99]
Combination	10 (14.9%)	[34,35,64,66,70,73,76,77,79,81]
Electronic	6 (9.0%)	[32,44,52,63,93,95]
Unclear	30 (44.8%)	[36,37,40,41,42,43,45,46,48,50,51,53,54,56,58,67,68,69,72,84,86,87,88,89,90,91,92,94,96,97]
**Study design**		
Prospective observational (no control)	25 (37.3%)	[35,41,50,52,53,56,58,62,63,64,66,72,73,75,76,77,78,79,80,84,95,96,97,98,99]
Pre and post comparison (with control)	21 (31.3%)	[34,36,37,38,39,42,43,44,45,47,48,49,51,55,57,60,61,86,88,91,93,94]
Prospective observational (with control)	13 (19.4%)	[46,50,53,54,59,69,81,82,85,89,90,92,95]
Randomized control trial	8 (11.9%)	[40,65,67,68,70,71,74,87]

^1^Includes two or more disease sites; ^2^Includes ovarian, cervical, vaginal and/or endometrial cancer; ^3^The integrated care plan was implemented in multiple countries simultaneously.

### Thematic analysis

The key themes that emerged from the thematic analysis are organized into five broad categories:

1. Design: Inputs associated with the design of integrated care plans.
2. Components: Core features that were observed across different types of integrated care plans.
3. Outcome measurement: Indicators/instruments used to measure the impact of integrated care plans.
4. Facilitators: Key factors that support the design, uptake and implementation of integrated care plans.
5. Barriers: Challenges associated with the development and use of integrated care plans.

**Figure 2:**
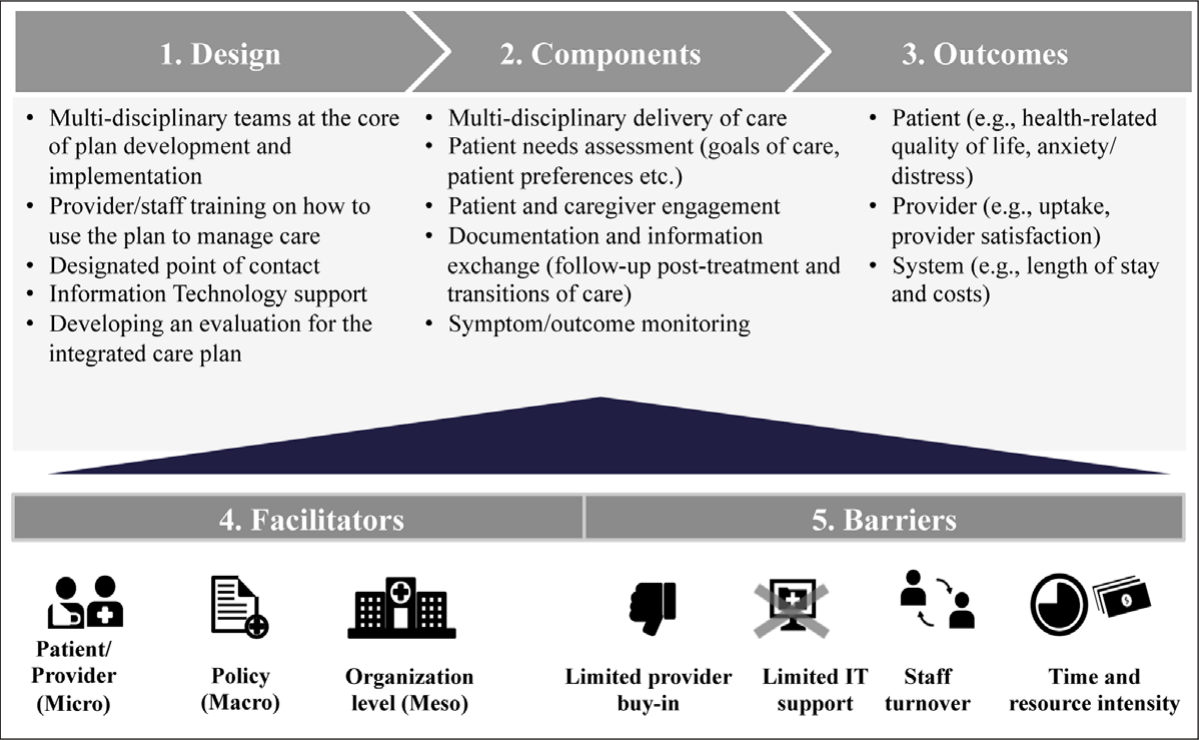
Integrated care planning for cancer care framework.

These themes are depicted in the integrated care planning for cancer care framework available in Figure 2.

#### Design features

Multi-disciplinary teams were at the forefront of designing and implementing integrated care plans. Providers from multiple disciplines were engaged to examine gaps in existing care, which then served as the impetus for developing the integrated care plan [60]. Patient involvement in the design phases was quite limited, with only a few studies reporting a targeted effort to engage patients at the outset to better understand and incorporate patient perspectives into plan development [64,65,71]. Overall, the development of integrated care plans appeared to be an iterative process; typically involving revisions over time and some type of impact assessment to assess the effectiveness of the integrated care plan [38,48,50,85,93], which was typically led by a specific team or provider tasked with examining uptake and outcomes [62,94,95].

Training for providers/staff on how to use the integrated care plan prior to full-scale implementation was seen as vital to enhancing buy-in around the use of the integrated care plan as a tool to enhance quality of care [34,64,82,85,86,87,91,93]. Training sessions were often customized to the healthcare team and procedures native to their work environments [47], and conducted through in-person, phone or web-based sessions [82,86]. Ensuring that the integrated care plan could be adapted to meet evolving patient’s needs [65,70] and was aligned with operational procedures at the organizational and provider-level also emerged as a common feature of the design phase across the different types of integrated care plans [48,78,91,96].

Having an implementation point of contact, i.e., a provider tasked with supporting plan uptake, was another key design feature of integrated care plans [85]. This typically ranged from a single member of the development team such as a nurse offering onsite supervision for frontline staff [85] to a broader committee tasked with supporting data collection and evaluation efforts [57,95]. Integrated care plans were often linked to the patient’s electronic medical record and could be reviewed by multiple providers [34,35,61,76,77]. Information technology capacities to efficiently generate the integrated care plan from the patient’s electronic medical record was another key design element [71,73,95], especially in articles that were focused on the survivorship stage of care [70,71].

#### Components

Across the various types of integrated care plans, care was organized and delivered by multi-disciplinary teams, involving providers from two or more disciplines. Disciplines represented included physicians, oncologists, surgeons, specialists, and primary care physicians, nurses (including specialist nurses and nurse navigators), pharmacists and allied health professionals such as social workers, speech pathologists, nutritionists/dieticians, as well as respiratory and physical therapists.

An assessment of patient needs was observed across the various types of integrated care plans. Within survivorship care plans, psychosocial distress [70] and quality of life [68,69] were commonly assessed to help identify psychosocial needs [60] and connect patient’s with appropriate providers, involving either hospital and/or community-based follow-up to obtain nutritional advice, spiritual care and financial counseling [70,84]. Other types of patient assessments include evaluation by a dietician/nutritionist [50,52,84] or speech pathologist (following surgical intervention) [52], or an evaluation of symptoms following treatment or at the end-of-life [50,63,85].

The establishment of goals of care that inform activities delivered within the plan was observed in integrated care plans focused on the survivorship or palliative stages of care. Although the nature of goal setting varied considerably. For example, in integrated care plans focused on the palliative stage of care, the provider examines the patient and/or family’s preferences, typically clarifies the goals of care with patients/caregivers, and then generates a treatment plan to align with those preferences [93]. In contrast, goal-setting in the survivorship phase was more focused on behavior and lifestyle change such as better nutrition and exercise to improve long-term health following cancer treatment [35,77].

Organizing follow-up care post treatment was commonly observed in integrated care plans focused on the surgical and survivorship stages of care. Within integrated care plans focused on surgical care, the emphasis was on short-term recovery from the procedure (i.e., wound care and dietary restrictions) and scheduling post-operative consultations [45,50,58,97]. Whereas among integrated care plans that were focused on survivorship, follow-up care involved educating patients on the appropriate recommendations for future screening, counseling on lifestyle changes, i.e., diet, exercise, smoking cessation, and links to resources for psychosocial, financial and sexual health issues [81].

Across the different types of integrated care plans there was a strong emphasis on the sharing of information within teams (i.e., between staff such as surgeons, nurses and allied health professionals within hospitals) and across organizations e.g., between hospitals and primary care practices involving hospital staff that are coordinating the discharge and transition of care to family physicians. Information exchange via integrated care plans often included aspects such as cancer type/stage, treatment, prognosis, side effects, medication lists prior to discharge, and follow-up care with oncology providers, surgeons and/or family physicians [34,36,45,48,50,51,55,56,96]. The nature of information exchange ranged from a summary of the patient’s treatment and symptoms being shared across providers via post, electronically [70,71,76,80,93], or fax [64] to a comprehensive multi-page plan which included information on symptoms and side effects, psychosocial needs, and community-based supports for the patient as they transition into survivorship [39,74].

Transitional care planning was another prominent feature especially among integrated care plans focused on the surgical stage of care, and included activities such as discharge planning [36,48,52,60] and referrals to external organizations/providers to manage post-treatment needs [36,56,59,64]. Integrated care plans were often shared with other providers at key transition points – particularly when patients moved from one stage of care to another, specifically from active treatment into survivorship [36,48,55,59,64,94].

Symptom or outcome monitoring, i.e., a feedback loop to inform changes to the plan was observed primarily in integrated care plans targeted towards the surgical and palliative stages of care [50,85,91,92,100], as well as systemic treatment [99]. A strong emphasis on documentation of care, was observed in integrated care plans that were focused on surgical or survivorship stages, as a means to identify gaps in adherence to the plan [59,62,65,77,85]. This particular aspect of integrated care plans was seen as a useful mechanism in promoting role clarity between different providers over the course of the patient’s journey [60], examining variance in adherence to a specific sequence of care activities [34,38,59,60,61,90], and promoting accountability across providers, and between patients and providers [61].

Patient/caregiver engagement by way of involving patients in generating the integrated care plan and accounting for patient (and/or caregiver preferences) featured prominently in plans focused on survivorship and palliative stages [43,59,62,63,78]. Some studies also reported allowing patients to access the integrated care plan through an electronic or paper format to enable patients to be actively engaged in treatment planning and delivery during their cancer journey [35,59,77].

The designation of a key point of contact for patients (during and following treatment) was also observed in some articles [42,53,62,97]. That role was usually held by a nurse who would typically connect with the patient via phone following treatment/discharge from hospital and also arrange follow up care [42,53,59,62,97]. Patient/caregiver education (oral and written) e.g., self-management support, nutritional counseling, information about homecare, wound care, health promotion (smoking cessation and exercise) [36,40,42,55,62,100], and referrals to cancer support groups [64] were other components of integrated care plans – especially those focused on survivorship care.

#### Outcome measurement

Broadly the outcomes used to assess the impact of integrated care plans can be categorized at three-levels: patient, provider and the system-level outcomes. Patient and system-level indicators were commonly reported whereas only a few studies examined provider-level outcomes.

Measurement approaches for patient-level indicators were typically drawn from validated tools, i.e., Impact of Events Scale [65] and the Short Form 36 Questionnaire [46,63,65,67]. In contrast, outcome measurement was considerably less robust for provider-focused outcomes. Studies that examined provider-level outcomes often did so primarily through a qualitative lens. Examples include assessing provider satisfaction through interviews [66,78], ascertaining integrated care plan uptake via chart reviews [93], using self-report methods for indicators such as time required to complete the integrated care plan [80]. An overview of the measurement tools used to assess the impact of integrated care plans on patient, provider and system-level outcomes is available in Table 3.

**Table 3:** Overview of measurement tools used to assess the impact of Integrated Care Plans.

Indicators	Measurement tool/instrument

**PATIENT**	

**Quality of life**	• Short Form 36 Questionnaire [45]
	• Short Form 12 [46]
	• European Organization for Research and treatment of Cancer Quality-of-life questionnaire [46,64]
**Patient satisfaction**	• Medical Outcomes Study – Patient Satisfaction Questionnaire [65]
	• System Usability Scale (modified) [80]
**Anxiety/distress**	• Spielberger State-Trait Anxiety Inventory [45]
	• Brief Symptom Inventory [64]
	• Cancer Survivors Unmet Needs Scale [64]
	• Impact of Events Scale [65]
	• Profile of Mood States [65]
	• Distress Thermometer [71]
	• Patient-Perceived Coordination Index [94]
	• Hospital Anxiety and Depression Scale [72]
**Caregiver-reported outcomes**	• Toolkit After-Death Family Member Interview [86]
	• Views of Informal Carers Evaluation of Service Survey [86,88)
	• Evaluating Care and Health Outcomes for the Dying [92]
	• Family Satisfaction Survey [93]
**PROVIDER**	

**Uptake**	• Chart reviews/retrospective audit [93]
**Workflow – Time to complete care plan**	• Provider self-report [80]
**Provider satisfaction**	• Telephone interviews [86,93]
	• System Usability Scale (modified) [80]
	• Consumer Assessment of Healthcare Providers and Systems Adult Specialty Care Clinician Questionnaire (modified) [80]
SYSTEM*	

**Length of stay**	• Number of nights spent in the hospital after surgery
**Post-operative complications**	• Post-operative complication rates
**Mortality**	• In-hospital mortality
**Readmissions**	• Hospital readmissions
**Costs**	• Total costs of hospital stay
	• Total cost of delivering the plan
	• Cost-effectiveness (i.e., quality adjusted life years gained for cost incurred)

*Since most system-level indicators represent standardized metrics individual references are not provided.

Commonly observed patient-level outcomes included patient satisfaction, cancer-related distress, patient perceptions of care coordination, patient-reported provider knowledge around the effects of cancer on survivors, perceived knowledge about survivorship [82] and Health-related Quality of Life. Caregiver experience was often examined as an outcome indicator for integrated care plans focused on the palliative stage of care [87,92]. Provider-level outcomes included provider satisfaction with the use of integrated care plans, i.e., workflow interruptions, ease of use, time associated with generating or discussing the integrated care plan with patients [74,83], and provider’s perceptions of benefits (i.e., improvements in information sharing between providers) [71,74,79,80,83,98]. System-level indicators included common healthcare utilization measures such as length of stay, complication rates (post-operative), hospital readmissions, and cost (hospital-related costs and the cost of delivering the integrated care plan) [36,39,40,41,43,46,47,48,49,51,54,55,56,57,58,60,61,67,85,95].

At a patient-level, the impact of integrated care plans was fairly mixed. Some studies reported improvements in patient satisfaction [45,52,60,82,95], patient involvement and patient-reported anxiety (at discharge) [45]. Whereas, no statistically significant differences were observed in cancer-related stress/distress [65], quality of life, and patient satisfaction [65,74], pre-operative anxiety levels [45] and Health-related Quality of Life [46]. Other trends included improvements in patient awareness about their cancer treatment and side-effects [74,83,98], knowing which provider is responsible for follow-up care, and greater patient involvement in managing end-of life care (specifically around the decision to use potentially life-shortening drugs to alleviate symptoms) [89]. One article found statistically significant improvements in patient perceptions around coordination between providers following the use of an integrated care plan to improve survivorship care, but no change in the extent to which the integrated care plan affected how providers understood a patient’s medical history and its implications on their quality of life [82].

At the provider-level, there were self-reported improvements in provider capacity to better manage their patients, specifically in the context of symptom burden [88,90,91,93,95], and better communication between providers [35,70,73]. Since provider-level measures were seldom reported and typically did not involve the use of standardized indicators, trends in provider-level outcomes could not be examined across articles.

System-level indicators were measured primarily in integrated care plans that focused on the surgical phase of care. Overall, general trends around changes in outcomes following the use of integrated care plans revealed improvements in system-level indicators, in particular decreased length of stay [34,36,37,38,41,42,44,46,47,50,57,60,61,62], reduced costs (direct costs associated with hospital stay) [34,38,39,42,44,57,60,62,95], decreased post-operative complication and lower readmission rates [41,55] or no change in costs [58]. One study found that the use of the integrated care plan was associated with a small increase in costs (with total quality-adjusted life years being almost equivalent between the intervention and control group) [67].

Several articles reported on the use of an existing care plan – primarily the LIVESTRONG, Journey Forward or Liverpool Care Plan respectively. Most study designs involving the Liverpool Care Pathway were either randomized control trials or had a comparable control group. One study reported statistically significant improvement in the extent to which patients were treated with respect, kindness and dignity, and the degree to which family emotional support, family self-efficacy, and coordination of care, but found no significant improvements in symptom control [86]. Whereas other studies indicated no differences in outcomes between patients cared for using the Liverpool Care Pathway and controls [87,92]. Other trends involving the Liverpool Care Pathway included a decrease in patients receiving potentially life-shortening drugs to alleviate symptoms [89], and reductions in uncontrolled symptoms at death [91].

Findings associated with the LIVESTRONG Care Plan were mixed. One article reported no changes in patient-reported distress, quality of life, and patient satisfaction [74], while another indicated over 90% of users reporting that the information provided by the integrated care plan was excellent, very good, or good (n = 276), with a further 60% of users reporting that the plan offered information that had not been offered by providers (n = 186), and 61% of users reported that the plan had enabled them to be more engaged with their healthcare team [32]. A majority of users also indicated that the LIVESTRONG Care Plan had increased their knowledge of potential long-term impacts of their cancer and what follow-up tests were required as they enter the survivorship phase [32].

For the Journey Forward Care Plan – one study reported that a majority of respondents found the plan to be very useful [78]. And in a second study among patients with breast cancer, over 70% of patients (n = 36) reported that the plan provided clarity around the importance of follow-up visits to check for late effects of cancer treatment, and helped them to better understand the value of engaging in health-promotion behaviors following treatment [76].

#### Facilitators

Key facilitators that enable and support the development and on-going use of integrated care plans were conceived to occur at three levels:

1. Micro: Patient and provider-level factors that support integrated care plan development and uptake
  Provider involvement in plan development and buy-in was a crucial facilitator [57,84,90,91,94], by way of helping to foster a sense of ownership and accountability over the integrated care plan’s value [57]. Engaging a diverse range of providers during the development phase may help ensure that the plan is customized to existing workflows and variations in scope of practice across provider disciplines, and supports greater transparency in role clarity and accountability between providers. Moreover, involving patients and caregivers as a means to empower them as participants in their own care to promote transparency between patients and providers was also highlighted as an important enabler [64].
2. Meso: Structural or process elements at the organizational level
  A dedicated provider or team that oversees integrated care plan development and implementation by serving as a conduit between providers and organizational management, and advocates for plan uptake was described as an important facilitator [57,62,85,86]. Information technology capacities to enable patients and providers to access the integrated care plan [73,78,93] and the alignment of integrated care plan use with existing workflows also served as important meso-level facilitators.
3. Macro: Policy-level factors that support the design and uptake of integrated care plans
  National oncological care policies that establish or promote standards around treatment guidelines and care management provide a yardstick for quality around patient care [96] and create a supportive environment for plan uptake. Other macro-level facilitators included incentivizing uptake by minimizing administrative barriers (i.e., process of reimbursement for plan use) that providers may face in trying to promote the integrated care plan as the standard of care [77,80].

#### Barriers

The barriers associated with use of integrated care plans originated mostly at the provider level and included most prominently limited provider buy-in and a lack of physician leadership [39,93]. In one study, the lack of provider buy in was attributed to medical education, specifically that palliative care is not part of undergraduate medical training in Italy, so physicians may not consider arranging palliative care to be within their scope of practice [87]. Other challenges included provider reluctance (and the learning curve) associated with adopting a new approach towards organizing care and misalignment between activities in the plan and existing workflows [63,85,93], Staff turnover also inhibited integrated care plans from becoming embedded in workflows as the standard of practice [59,94,95].

Inadequate information technology support, specifically limited functionality of the electronic medical record in generating an integrated care plan for survivorship care was an important barrier due to the time and resource costs associated with providers manually entering items from the health record rather than an automated transfer [79]. In addition, difficulties in accessing integrated care plans from medical records across organizations [63], limited co-location of providers, and inadequate financial compensation for using integrated care plans [64,73,78,80] were among other reported barriers to use [63].

## Discussion

While there is a growing emphasis on the use of care plans to guide the organization and delivery of care for cancer patients, our understanding of the components and key facilitators of integrated care plan uptake is still in its early stages. This review was unable to identify a single integrated care plan that spanned across all stages of the cancer journey from diagnosis through to survivorship or palliative care. From the articles that were included, most integrated care plans appear to be focused on a single stage. Integrated care plans that span across two stages of the cancer journey (i.e., surgical to survivorship care) represent less than 10% of included articles, indicating that transitions of care have not heavily featured as a core attribute of integrated care delivery for cancer patients, and that this gap requires further exploration [1]. Most of the integrated care plans reviewed were focused on the surgical stage of care, which reflects a general trend around the use of care planning methodologies to primarily guide clinical practice (typically in acute care settings) rather than being employed across healthcare settings along the continuum of care for a specific patient group or illness [101,102,103]. The disproportionate use of integrated care plans for surgical stages of care is in line with a historically strong emphasis on using clinical outcomes to assess the impact of integrated care plans, clinical pathways etc., observed in current literature [18,101,102,103]. And in fact, Van Herck et al. found that clinically-oriented outcomes were assessed in 65.5% of articles they reviewed in assessing the effectiveness of clinical pathways, whereas team and service effects received considerably less attention [18].

Similarities were however noted across the different types of integrated care plans included in this review in terms of the design features and core components observed, indicating the potential to leverage these shared elements to create an integrated care plan that spans across stages of the cancer journey, including key transition points [1,3].

The adoption of an integrated care plan to guide the delivery of care relies heavily on provider uptake and training on how to use the integrated care plan [34,39,43,60,61,75,94]. The upstream involvement and continuous engagement of providers during plan development and evaluation were considered core elements of the design phase [38,39,60]. Provider training, oversight from a dedicated provider-based committee and program champions have been previously recognized as important facilitators for the uptake and on-going use of integrated care plans or clinical pathways (and related concepts) in other studies [104,105].

Many elements of the integrated care plan development process, including a clear identification of a target population, multi-disciplinary development, sequencing of care activities, and staff training map onto key elements of existing pathway development methodology [104,106]. However this formal process/approach towards plan development was not always clearly articulated in the articles included in this review.

Many of the patient-level metrics reported across articles reflect indicators and instruments that are increasingly categorized as patient-reported outcome measures in cancer care, including the European Organization for Research and Treatment of Cancer Quality of Life Questionnaire, Brief Symptom Inventory, Functional Assessment of Cancer Therapy-G, Profile of Mood States, and the Distress Thermometer [107]. Interestingly, while integrated care plans are intended to be patient-facing, patient engagement in the development phase was reported by only a handful of articles [64,65,71]. This disconnect highlights the need to involve patients at the outset of plan development, since integrated care plans are intended to be patient-facing tools, particularly in light of growing emphasis on patient engagement and the inclusion of patient-reported outcomes in assessing the quality of cancer care more recently [107,108,109,110,111].

Compared to patient and system-level indicators, outcome measurement at the provider level was less robust, with only a handful of studies reporting on provider experiences with using integrated care plans to deliver care. Since providers are core end-users of integrated care plans, uptake relies heavily on provider commitment, and it is surprising to see a prominent gap in the assessment of provider-focused outcomes across different types of integrated care plans included in this review. In addition, findings also revealed limited reporting on process indicators, highlighting the need for greater attention towards developing indicators intended to help understand how integrated care plans affect team functioning (within and across healthcare settings) and inform the operational aspects of care delivery as it relates to cancer care.

System-level outcome trends emerging mainly from integrated care plans focused on surgical treatment indicate reductions in length of stay [34,36,37,38,42,46,47,50,57,61,62], costs (direct costs associated with hospital stay) [34,38,39,42,57,62,95] and post-operative complication rates [55]. Changes in patient-level outcomes were mixed; some studies indicated improvements in patient satisfaction [45,60,82,95] and patient-reported anxiety [45], whereas others report no statistically significant differences in indicators such as cancer-related stress] [65] and Health-related Quality of Life [46]. Since study quality is not accounted for in scoping reviews it is not possible to draw reliable conclusions around the outcomes of integrated care plans from the trends observed. Overall the current state of knowledge around the outcomes associated with care plans for cancer patients is still in its early stage [22]. More work is needed to advance our understanding of the short and long-term outcomes associated with using integrated care plans to support the organization and delivery of care for cancer patients.

## Limitations

One of the key limitations of this work is the restricted extent to which generalizations can be made about the impact of integrated care plans in terms of patient, provider and system-level outcomes. Since study quality including sample size and study design was not accounted for, findings represent a description of broad themes observed across articles. In addition the absence of a common and generally accepted definition of an integrated care plan, and a lack of consensus around the attributes of integrated care delivery as it pertains to cancer care, may have narrowed the breadth of articles that were captured in this review. Nonetheless, efforts were made to ensure that the search terms used to guide this scoping review enabled us to capture a wide range of constructs, and the search strategy was validated by the project team and with an external librarian.

Another limitation is the inclusion of articles that involved the development and pilot testing of an integrated care plan, as long as the outcomes associated with pilot-testing were discussed in the article, which may not reflect the outcomes or challenges associated with full-scale implementation. In addition, authors often did not report on key operational details (e.g., organizational inputs or process measures etc.) associated with developing and/or implementing integrated care plans. And most studies did not report on patient characteristics (aside from site and stage), thereby the complexity of the patient population including multi-morbidity, socio-economic status, and caregiver support, and its potential impact on the uptake and effectiveness of integrated care plans is hard to quantify.

## Conclusion

Findings indicate that an integrated approach to care delivery over the course of the cancer journey is still in its early stages. Similarities in design features, core components and key facilitators across the various types of integrated care plans highlight an opportunity to leverage these shared features to move towards developing integrated care plans that span the course of a patient’s cancer journey, rather than a phase-specific silo approach that currently dictates the way care is delivered.

Multi-disciplinary teams, iterative development, patient needs assessment, and transitional planning emerged as key features of integrated care plans for cancer patients. Gaps in information technology support, limited physician buy-in as well as time and resource intensity were identified as key barriers to plan uptake, and must be considered at the outset of plan development. In terms of outcome measurement, patient and system-level indicators were robustly assessed, whereas provider-level metrics were less commonly explored and typically did not involve the use of a validated measurement instruments etc. Further exploring how outcomes vary across disease sites and patient sub-groups, and expanding our knowledge base around measuring patient and provider-level outcomes are important next steps to consider. Future work on this topic is warranted, in particular validating the conceptual framework through broader expert consensus, and developing and piloting an integrated care plan in partnership with providers, patients and administrators that work in the cancer system along with other key stakeholders including primary and community care providers.
